# Output Regulation and Function Optimization of Mitochondria in Eukaryotes

**DOI:** 10.3389/fcell.2020.598112

**Published:** 2020-11-17

**Authors:** Miaolin Zeng, Yu He, Haixia Du, Jiehong Yang, Haitong Wan

**Affiliations:** ^1^College of Basic Medical Science, Zhejiang Chinese Medical University, Hangzhou, China; ^2^College of Pharmaceutical Science, Zhejiang Chinese Medical University, Hangzhou, China; ^3^College of Life Science, Zhejiang Chinese Medical University, Hangzhou, China

**Keywords:** mitochondria, eukaryotes, metabolism, mitochondrial transfer, mitochondrial dynamics, mitophagy, apoptosis

## Abstract

The emergence of endosymbiosis between aerobic alpha-proteobacterium and anaerobic eukaryotic cell precursors opened the chapter of eukaryotic evolution. Multiple functions of mitochondria originated from the ancient precursors of mitochondria and underwent remodeling in eukaryotic cells. Due to the dependence on mitochondrial functions, eukaryotic cells need to constantly adjust mitochondrial output based on energy demand and cellular stress. Meanwhile, eukaryotes conduct the metabolic cooperation between different cells through the involvement of mitochondria. Under some conditions, mitochondria might also be transferred to nearby cells to provide a protective mechanism. However, the endosymbiont relationship determines the existence of various types of mitochondrial injury, such as proteotoxic stress, mutational meltdown, oxidative injure, and immune activation caused by released mitochondrial contents. Eukaryotes have a repertoire of mitochondrial optimization processes, including various mitochondrial quality-control proteins, regulation of mitochondrial dynamics and activation of mitochondrial autophagy. When these quality-control processes fail, eukaryotic cells can activate apoptosis to intercept uncontrolled cell death, thereby minimizing the damage to extracellular tissue. In this review, we describe the intracellular and extracellular context-based regulation of mitochondrial output in eukaryotic cells, and introduce new findings on multifaceted quality-control processes to deal with mitochondrial defects.

## Introduction

The great oxygenation event (GOE), which occurred 2.3–2.4 billion years ago, created a permanently oxygen-containing atmosphere on Earth, initiating the development of aerobic organisms (Rantamaki et al., 2016). The subsequent emergence of endosymbiosis between aerobic alpha-proteobacterium (the precursor of mitochondria) and anaerobic eukaryotic cell precursors greatly accelerated eukaryotic evolution (Sagan, 1967; Roger et al., 2017). The combination of a more elaborate genetic organization pattern and a remarkably efficient energy metabolism ultimately allowed eukaryotes to achieve a complex biodiversity that prokaryotes could not match.

Exchange of metabolites due to complementary metabolic patterns determined by different genetic backgrounds is presumed to be the main driver of this cross-species alliance (Morris, 2015; Estrela et al., 2016). The symbiont gained a more stable source of energy substrates from the host, as well as a safer shelter and greater transport capacity (Johnson and Bronstein, 2019). Eukaryotes provided the symbiont with metabolites that eukaryotes could not further utilize in exchange for enormous energy, making the alliance a powerful force in natural competition (Ghoul and Mitri, 2016). As endosymbiosis became more interdependent, eukaryotes began to exert a central control over the symbiont, including nuclear transfer of the symbiont genes, formation of a mechanism to import proteins from the host cytoplasm to the symbiont, cell division synchronization between host and symbiont, marking the beginning of an obligate and irreversible partnership (Uchiumi et al., 2019).

Now, as the ultimate model of this endosymbiont, eukaryotes can manipulate the behavior of mitochondria to maximize their output/investment ratio and shape mitochondria based on intracellular and extracellular context. Eukaryotes regulate ATP production and metabolic support of mitochondria by regulating the metabolic coupling status of cytoplasm and mitochondria, so as to cope with the constantly changing metabolic demand and cellular stress. Eukaryotes also conduct the metabolic cooperation between different cells through the involvement of mitochondria. Under some conditions, mitochondria might also be transferred to nearby cells, thus providing a protective mechanism. Meanwhile, eukaryotes also need to address the remaining defects in this endosymbiotic relationship, such as proteotoxic stress, mutational meltdown, oxidative stress, and inflammatory activation caused by the release of mitochondrial contents. Eukaryotes have a repertoire of mitochondrial quality optimization processes, including various mitochondrial quality-control proteins, regulation of mitochondrial dynamics and activation of mitophagy. Under relatively mild stress, adaptive responses in the mitochondria help to restore homeostasis. Conversely, when the damage is irreversible and exceeds the cell’s compensation, the release of substances in mitochondria caused by danger signals can be a means of inducing apoptosis. When ATP is sufficient, apoptosis almost always takes precedence over necrosis, a more uncontrolled form of cell death, thus minimizing damage to external tissues caused by the release of mitochondrial contents due to cell membrane rupture.

Here, we discuss the intracellular and extracellular context-based regulation of mitochondrial function in eukaryotic cells. The emerging knowledge about multifaceted quality-control processes to deal with various mitochondrial defects will also be described.

## Metabolic Coupling Between Cytoplasm and Mitochondria

**FIGURE 1:**
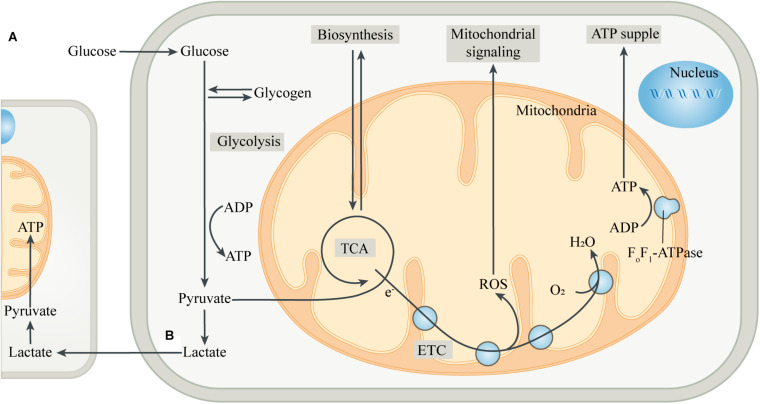
Intracellular metabolic coupling and intercellular metabolic cooperation. **(A)** The coupling of glycolysis of the cytoplasm with aerobic metabolic processes of mitochondria constitutes the center of cellular energy metabolism. Meanwhile, mitochondria provide many substrates for cell anabolism, and the by-products produced by mitochondria are important signaling substances. **(B)** Pyruvate, produced by glycolysis, is partially converted to lactate, which is then released into the extracellular space and ingested by adjacent or distant cells. After entering the cell, lactate is converted to pyruvate, which is then transferred into the mitochondria and is oxidized by mitochondria for energy, thus completing the metabolic cooperation between cells. Abbreviations: ETC, electron transport chain; TCA, tricarboxylic acid cycle.

The incorporation of mitochondria led to dramatic changes in the metabolic patterns of eukaryotes. The metabolic differences caused by different genetic backgrounds drive the coupling of metabolism between eukaryotic cells and mitochondria, greatly improving the efficiency of metabolic substrates to produce ATP (Morris, 2015; Estrela et al., 2016; Figure 1). Most eukaryotic cells use glucose transporters to absorb glucose from the blood or surrounding tissues as the preferred substrate for energy metabolism. In the absence of mitochondria, glucose produces energy in the cytoplasm through glycolysis. Each glucose molecule is catalyzed by a series of glycolytic enzymes to produce 2 pyruvate molecules and 2 ATP molecules, with NAD^+^ being reduced to NADH (Magistretti and Allaman, 2018). Pyruvate is then converted to lactate by lactate dehydrogenase (LDH), and the process does not produce ATP, but leads to the regeneration of NAD^+^, which is used repeatedly to drive glycolysis. Then, lactate is transported to the extracellular space through monocarboxylate transporters, pannexins or some ion channels (Sotelo-Hitschfeld et al., 2015; Karagiannis et al., 2016; Elizondo-Vega and Garcia-Robles, 2017). However, in most cell types, pyruvate produced by glycolysis preferentially enters the mitochondria as a substrate of oxidative phosphorylation (OXPHOS) in the presence of mitochondria.

After entering the mitochondrial matrix compartment, pyruvate is first converted to acetyl-CoA by pyruvate dehydrogenase (PDH). In the tricarboxylic acid cycle (TCA cycle), acetyl-CoA is gradually broken down by a series of enzymes to produce carbon dioxide, with the electrons directly or through other electron carriers (NADH and FADH_2_) in the matrix being transferred to the coenzyme Q pool in the mitochondrial inner membrane (MIM) (Smith et al., 2012). The electrons then pass through complex III and cytochrome C, respectively, and finally combine with the oxygen in complex IV to form water. During the transmission of the electron transport chain (ETC), protons are pumped from the mitochondrial matrix into the mitochondrial intermembrane space (MIMS), to form a proton motive force, which is composed of a transmembrane potential and a pH gradient (Murphy and Hartley, 2018). The proton motive force ultimately promotes the conversion of ADP to ATP by turbine-like F_0_F_1_-ATPase or complex V (Nicholls et al., 2015). The “assembly line” of glycolysis and mitochondrial oxidation produces a net 32 ATP molecules from one glucose molecule, which is significantly more efficient than glycolysis alone. The produced ATP is transported to the cytoplasm through the adenine nucleotide translocase (ANT) and used by the cell (Palmieri, 2013). The mitochondrial membrane potential generated by OXPHOS also has a variety of functions, such as providing driving force for mitochondrial protein transmembrane transport, sensing the cell’s internal and external environment, and participating in the regulation of mitochondrial dynamics (Neupert and Herrmann, 2007; Gilkerson et al., 2012). In addition, mitochondria can import other energy substrates from the cytoplasm such as amino acids and fatty acids, and utilize them to fuel the TCA cycle when the supply of carbohydrate substrates is insufficient.

Mitochondria, in addition to serving as the hub for catabolism, also provide various substrates for cellular anabolism. The intermediates in the TCA cycle serve as precursors for the biosynthesis of many macromolecules, including lipids, carbohydrates and amino acids (Sibson et al., 1998; Waagepetersen et al., 2001). Mitochondria can also regulate the levels of a variety of cofactors, which control the activity of multiple intracellular enzymes, including DNA modification enzymes such as histone deacetylases (Lkhagva et al., 2018). Moreover, mitochondria are key sites for metal ion metabolism to synthesize heme and Fe-S clusters, and participate in some important biological activities such as ketogenesis, steroidogenesis, gluconeogenesis and ammonium detoxification (Baughman et al., 2011; Rouault, 2015). In addition to being involved in cellular bioenergetics and anabolism, mitochondria are also important signaling organelles. The TCA cycle produces substrates for the synthesis of neurotransmitters, such as glutamate, GABA and acetylcholine, thus forming the basis of synaptic signaling (Sibson et al., 1998; Waagepetersen et al., 2001). Reactive oxygen species (ROS), mainly produced in complex I and complex III of mitochondrial ETC, is an important cellular signal at moderate levels, while excessive ROS can injure cellular components (Schieber and Chandel, 2014). Mitochondria are also important organelles for regulating intracellular Ca^2+^ homeostasis, while the abnormal increase of Ca^2+^ in mitochondria can control the opening of mitochondrial permeability transition pore (mPTP), which induces cell apoptosis and is also an important factor for promoting necrosis (Orrenius et al., 2003; Panel et al., 2017).

## Mitochondria Link Metabolic Cooperation Between Cells

During hypoxia, restricted mitochondrial OXPHOS and compensatively up-regulated glycolysis result in more conversion of pyruvate to lactate, which leads to the accumulation of lactate in the tissues. In contrast to this compulsory situation, there is a physiological mismatch between glycolysis and mitochondrial OXPHOS in some cells (Brooks, 2007). When the energy metabolism of one type of cell is mainly through glycolysis, on the contrary, the energy metabolism of another type of cell can be mainly through mitochondrial OXPHOS. Interestingly, these two types of cells co-exist closely in some tissues and form a cooperative combination to exchange energy substrates (Figure 1). The most representative examples are intercellular energy coupling between neurons and astrocytes in the central nervous system (CNS), and that between “red” and “white” muscle fibers in skeletal muscle (Brooks, 2009; Magistretti and Allaman, 2018).

In the CNS, ATP consumed by neurons accounts for 80–90% of the total brain consumption, and mitochondrial OXPHOS is the main energy metabolism mode of neurons (Howarth et al., 2012). In contrast, when the brain activity increases, most of the increment in glucose intake occurs in astrocytes (Chuquet et al., 2010; Zimmer et al., 2017). In astrocytes, pyruvate produced by glycolysis is only partially oxidized by mitochondria, while most of it is used to produce lactate, which is then released and introduced into neighboring neurons (Magistretti and Allaman, 2018). Lactate is converted to pyruvate, which is oxidized by mitochondria to produce ATP to support neuronal activity. Neurons use lactate from astrocytes as a preferred substrate for mitochondrial OXPHOS, and the small amount of glucose they absorb is mainly used for a branch of glycolysis, the pentose phosphate pathway (PPP) (Herrero-Mendez et al., 2009). When neural activity increases, glutamatergic neurons up-regulate the synthesis and release of the neurotransmitter glutamate in the presynaptic membrane. In addition to stimulating the postsynaptic membrane to transmit nerve signals, the glutamate in the synaptic cleft can also trigger an increase in glucose uptake and lactate production in astrocytes, thus achieving an activity-dependent neuron-astrocyte metabolic coupling (Bolanos, 2016). Glutamate is also absorbed by astrocytes and, if necessary, can be converted to α-ketoglutaric acid (α-KG) by glutamate dehydrogenase (GDH) in the mitochondria, which then enters the TCA cycle to produce ATP.

Similar intercellular metabolic cooperation occurs in skeletal muscle, where the energy metabolism of “white” fast fibers is dominated by glycolysis, while that of “red” slow fibers is mainly by mitochondrial OXPHOS (Brooks, 2009). During intense exercise, the activated sympathetic nerve stimulates muscle glycogenolysis and recruits the fast-glycolytic fibers, which dominate short-term burst exercise, leading to a significant increase in lactate flux. ATP produced by glycolysis is the main energy source for the muscle contraction of fast fibers, and the large amount of lactate produced is transported to nearby slow fibers rich in myoglobin and capillaries (Reis and Wooten, 1970). Lactate is then converted to pyruvate, which is oxidized by mitochondria for energy to support the activity of the “red” slow fibers. Not all lactate produced in the working muscle bed is oxidized immediately. Unconsumed lactate is released into the blood stream as a substrate for energy metabolism in the heart, brain, kidneys, and other organs, or gluconeogenesis in the liver (Brooks, 2018).

Cell-specific metabolic profiles due to different genetic backgrounds determine the necessity of metabolic exchange between cells (Magistretti and Allaman, 2015). As the end product of glycolysis, lactate, with the participation of mitochondria, has become a tool to coordinate energy metabolism between neighboring or even distant cells, and to compensate for the unbalanced substrates supply and energy demand among different regions. Interestingly, these direct products or derivatives from energy metabolism, such as lactate, glutamate, GABA and acetylcholine, can be used as substrates for mitochondrial OXPHOS under certain conditions, and are also important signal molecules that mediate cell-to-cell communication (Newman et al., 2011; McKenna et al., 2016). This perfectly reflects the notion that energy metabolism is the core of life.

## Intercellular Mitochondrial Transfer Provides a Protective Mechanism

Mitochondria comprise the intracellular cores for energetics and viability, and mitochondrial dysfunction is an important pathogenesis of many diseases (Anne Stetler et al., 2013; Heyck et al., 2019). Recent studies suggested the presence of intercellular mitochondrial transfer in the body, which may influence the symptoms and functional outcomes of mitochondria-related diseases (Lippert and Borlongan, 2019; Montgomery, 2019). In a mouse model of transient focal cerebral ischemia, astrocytes released extracellular vesicles containing functional mitochondria (Hayakawa et al., 2016). These vesicles, which were between 300 and 1100 nm in size, were then ingested by nearby ischemic neurons. This crosstalk between neurons and astrocytes provided endogenous neuroprotection after stroke to maintain neuronal survival and promote the recovery of neurological function. Inhibition of CD38 with siRNA reduced the release of mitochondrial vesicles from astrocytes and worsened the prognosis of neurological function, suggesting that the release of extracellular vesicles from astrocytes is related to CD38/cyclic ADP ribose signal transduction. Another study found that the inhibition of mitochondria in astrocytes could accelerate the death of neurons, confirming that healthy mitochondria in astrocytes are an important part of the protective mechanism of astrocytes (Falchi et al., 2013). Interestingly, retinal neurons can transfer damaged mitochondria to astrocytes for disposal and recycling (Davis et al., 2014). This mitochondrial exchange serves as a way for neurons to remove dysfunctional mitochondria during stress and may represent a potential mode of intercellular signaling in the CNS.

In addition to mitochondrial transfer across cells through vesicles containing mitochondria, a newly identified membrane nanotubes (MNTs) structure exists between cells to act as a pathway for intercellular mitochondrial transport (Zhang and Zhang, 2013; Shen et al., 2018). MNTs are long, thin, membrane-based connections between cells that mediate the exchange of various substances (Rustom et al., 2004; Burtey et al., 2015). Studies have confirmed the existence of MNTs between ischemic cardiomyocytes (CMs) and cardiac myofibroblasts (MFs) to mediate mitochondrial transfer and promote cardiomyocyte survival by reducing apoptosis (Shen et al., 2018). In cell culture, treatment with microtubule polymerizing inhibitors nocodazole or colcemid, or knocking down the microtubule motor protein kinesin family member 5B (KIF5B) reduced mitochondrial movement, suggesting that intercellular mitochondrial transfer depends on microtubules and KIF5B. MNTs are also involved in Ca^2+^ transfer to participate in intercellular signaling.

Research on the treatment of mitochondria-related diseases by inducing mitochondrial transfer is emerging. In mice with acute lung injury (ALI) induced by airway infusion of lipopolysaccharide (LPS), airway administration of mouse bone marrow-derived stromal cells (mBMSCs) or human BMSCs (hBMSCs) reduced alveolar inflammatory infiltration, inhibition of surfactant secretion, and mortality in ALI mice (Islam et al., 2012). The mechanism is that BMSCs can form gap junction channels (GJCs) composed of connexin 43 (Cx43) with alveolar epithelium, and then release mitochondria-containing microvesicles, which are engulfed by alveolar epithelia. In transient middle cerebral artery occlusion (tMCAO) model rats, direct injection of mitochondria isolated from autologous pectoralis major into the lateral ventricles showed neuroprotective effects, reduced infarct volume, and improved neurological function at 4 weeks after stroke (Zhang et al., 2019). Mitochondrial transfer reduces oxidative stress and apoptosis, alleviates reactive astrogliosis, and promotes neurogenesis. Mesenchymal stem cells can also protect endothelial cells from ischemia reperfusion injury (IRI) via MNTs-mediated mitochondrial transfer (Liu et al., 2014). However, low transfer efficiency is a challenge in clinical settings. In the neonatal rat cardiomyocytes (NRCMs) with IRI, the use of transactivator of transcription dextran complex (TAT-dextran) enhanced the uptake of mitochondria into rat cardiomyocytes and protected the cardiomyocytes (Maeda et al., 2020). The enhanced transfer of mitochondria to NRCMs can significantly reduce the apoptosis of cardiomyocytes after oxidative stress and prevent the inhibition of OXPHOS in mitochondria.

## Quality Control Processes to Control Mitochondrial Proteotoxic Stress

One of the challenges presented to eukaryotes by the cross-species endosymbiosis is the regulation of gene expression from nuclear DNA and multiple copies of mitochondrial DNA (mtDNA). The four complexes of ETC involved in ATP synthesis in mitochondria are enzyme complexes composed of multiple subunits, all containing polypeptides encoded by genomes inside and outside organelles (Adams and Palmer, 2003). The mismatch of these proportionally assembled proteins can cause proteotoxic stress, manifested by protein accumulation and misfolding (Isaac et al., 2018). In addition, electrons sporadically escape from the ETC and form ROS, which can destroy nearby mitochondrial components, including nucleic acids, proteins, and lipids (Murphy, 2009). The highly activated acetyl-CoA in mitochondria can induce the acylation of non-enzymatic proteins, which can also exacerbate protein damage or misfolding in mitochondria (Trub and Hirschey, 2018).

Mitochondrial protein homeostasis is mainly dependent on a series of chaperones and proteases present in the organelles, and the quality control proteins in each individual compartment are different. In the matrix, the mitochondrial Hsp70, which is located near the translocase of the inner membrane (TIM), and its cofactor Hsp100, as well as the chaperonin complex consisting of Hsp60 and Hsp10, are dedicated to folding imported new proteins and refolding misfolded ones (Jensen and Jasper, 2014; Sorrentino et al., 2018). Mitochondrial proteases, including protease LON and ClpX/P in the matrix, AFG3L2/SPG7 on the inner surface of the MIM, Yme1 on the outer surface of the MIM, HtrA2 in the MIMS and Msp1/ATAD1 on the mitochondrial outer membrane (MOM), can degrade redundant, damaged or misfolded proteins (Koppen and Langer, 2007; Quiros et al., 2015; Wu et al., 2018). The recognition of targets by mitochondrial chaperones and proteases mainly depends on the sub-mitochondrial localization and topology of the proteins (Pareek et al., 2018; Sprenger et al., 2019).

**FIGURE 2:**
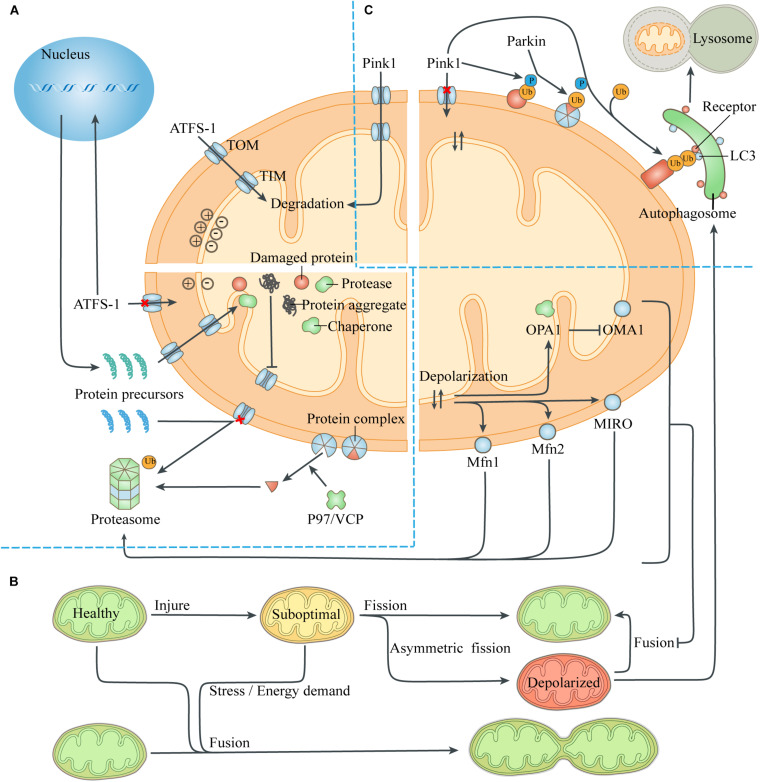
Quality-control processes to reduce the mitochondrial burdens. **(A)** Maintenance of mitochondrial proteostasis. The transcription factor ATFS-1 is routinely imported to the mitochondria and degraded. The abnormal mitochondrial membrane potential caused by damaged proteins and protein aggregates leads to the transfer of ATFS-1 into the nucleus instead of the mitochondria, resulting in increased expression of mitochondrial chaperones and proteases and their entry into the mitochondria. Mitochondrial proteotoxic stress blocks the input of nuclear-encoded mitochondrial proteins, which are then degraded by the ubiquitin-proteasome system. P97/VCP isolates damaged membrane-bound proteins or proteins in complexes, thus facilitating turnover of mitochondrial proteins exposed to the cytoplasm through the proteasome. **(B)** Mitochondrial fusion and fission. The exchange of components during mitochondrial fusion compensates for the defects of suboptimal mitochondria, thus maintaining ATP production and resisting stress. Asymmetric mitochondrial fission helps to isolate the damaged components in the mitochondria, which are removed by mitophagy. However, severely defective mitochondria could not fuse with other mitochondria due to the degradation of fusion-related proteins. **(C)** Mitophagy. The loss of mitochondrial membrane potential leads to the accumulation of the kinase Pink1 on the mitochondrial surface, which phosphorylates ubiquitin on the MOM proteins and recruits the E3 ligase Parkin. Parkin induces ubiquitination of more MOM proteins, which act as a platform to recruit modifier LC3 through different autophagic receptors. Then the autophagosome engulfs the mitochondria and fuses with lysosome to digest the mitochondria. Abbreviation: TOM, translocase of the outer membrane; TIM, translocase of the inner membrane.

When the pre-existing quality-control ability is exceeded, mitochondrial proteotoxic stress can directly inhibit mitochondrial translation and affect nuclear expression. This was termed unfolded protein response (UPR) and depends on the transcription factor ATFS-1 (Melber and Haynes, 2018; Boos et al., 2019; Figure 2). The presence of normal mitochondrial membrane potential can cause ATFS-1 to be routinely transported to the mitochondria and degraded by the protease LON (Nargund et al., 2012). When proteotoxic stress is severe, the decrease of mitochondrial membrane potential caused by mitochondrial dysfunction can prevent ATFS-1 from entering the mitochondria. Instead, ATFS-1 is transported to the nucleus and stimulates transcriptional up-regulation of genes encoding mitochondrial chaperones and proteases. Meanwhile, ClpP in mitochondria can be activated by excess unfolded proteins, and the pharmacological inhibition of protease LON can up-regulate the activity of Hsp60, indicating that the role of mitochondrial chaperones and proteases partially overlaps (Zhao et al., 2002; Munch and Harper, 2016).

Intracellular or extracellular stress can provoke a defective import of mitochondrial proteins, leading to the accumulation of protein aggregates in the cytoplasm, which are then degraded by the activated ubiquitin-proteasome system (UPS) (Bard et al., 2018). The presence of the MIM potential provides a driving force for the input of the mitochondrial protein precursors guided by the guiding peptide, and its decrease prevents mitochondrial proteins from entering the mitochondria. Moreover, The protease Msp1/ATAD1, embedded on the MOM, works with Cis1 to block the translocase of the outer membrane (TOM) and mediates the extraction of clogging proteins to the cytosol, when removing damaged or redundant proteins (Itakura et al., 2016; Wohlever et al., 2017). The 26S proteasome can only degrade soluble proteins, while the cofactor Cdc48 (mammalian P97/VCP) extracts substrates embedded on the MOM or bound to protein complexes, to help them be identified and removed (Weidberg and Amon, 2018; Youle, 2019).

In conclusion, in the presence of mitochondrial proteotoxic stress, eukaryotic cells promote chaperones to refold misfolded proteins and proteases to degrade damaged proteins by up-regulating the expression of corresponding nuclear genes. At the same time, the UPS in the cytoplasm is also involved in the clearance of abnormal proteins on the MOM to some extent. Multiple mechanisms work together to maintain proteostasis in mitochondria.

## Regulate Mitochondrial Dynamics to Optimize the Mitochondrial Population

Mitochondrial fusion and fission maintain the continuous changes of mitochondrial morphology, and also affect the number of mitochondria. Frequent mitochondrial fusion and fission allow different mitochondria to share genetic information and functional materials, thus resulting in a large, separate but functionally unified mitochondrial community. At the same time, the change of mitochondrial dynamics is helpful to maintain the mitochondrial energy output under stress conditions, and plays a purification role on the mitochondrial population.

Mitochondrial fusion and fission are controlled by the evolutionarily conserved large guanosine triphosphatases (GTPases), members of the dynamin family (Hoppins et al., 2007). Mitofusins (MFN1 and MFN2) on the MOM and optic atrophy protein 1 (OPA1) on the MIM control the fusion of MOM and MIM, respectively (Wai and Langer, 2016). Dynamin-related protein 1 (DRP1) recruited from the cytoplasm can spirally distribute around the organelles, gather and clip the two lipid bilayers of mitochondria, thus mediating mitochondrial fission (Sebastian et al., 2017). Eukaryotic cells can regulate mitochondrial fusion and fission by controlling the hydrolysis or post-translational modification of these proteins to meet energy requirements or stress.

Asexual reproduction and multiple copies of mtDNA put mitochondria at high risk of mutational meltdown. In mammals, mtDNA is almost matrilineally inherited, and its accumulated mutations can be removed during oocyte development, thus ensuring that mammalian offspring have a homogeneous and well-functioning mitochondrial genome at the beginning of life (Rojansky et al., 2016; Sebastian et al., 2017). However, it is unclear whether there is any quality control for mtDNA in somatic cells. The existence of multiple copies of mitochondrial genome leads to the lack of necessary connections between mutated mtDNA and defective protein products, providing difficulties for the mitochondrial quality control processes which mainly depends on mitochondrial function.

Mitochondrial fusion provides a means to dilute the concentration of mutated mtDNA genes and reduce the incidence of mitochondrial dysfunction. In the process of the fusion of mitochondria with mutant DNA and those with wild-type DNA, nucleoids in organelles were shared, thus reducing the proportion of total mtDNA defects (Nakada et al., 2001). Moreover, energy metabolism of mitochondria requires a highly accurate and well-functioning mitochondrial respiratory chain to ensure the production of ATP and reduce the accompanying ROS. When mitochondrial components are damaged by environmental injure, toxic stimulation or oxidative stress, mitochondrial fusion can compensate for individual defects by directly exchanging organelle components such as RNA, proteins and lipids, so as to realize complementary cooperation between mitochondria with different functional disorders (Schon and Gilkerson, 2010). Therefore, when cells face acute stress, mitochondrial fusion ensures the output of mitochondrial population and produces the maximum potential to resist stress.

Mitochondrial fusion can repair moderate organelle dysfunction, but multiple mechanisms can prevent severely damaged mitochondria from rejoining the mitochondrial network (Figure 2). The abnormal mitochondrial membrane potential and ATP deficiency caused by injury activate the protease OMA1 on the MIM, which hydrolyzes OPA1 and thus prevents MIM fusion (Head et al., 2009). Meanwhile, the abnormal membrane potential itself increases the difficulty of MIM fusion (Meeusen et al., 2006). Through the UPS, abnormal mitochondrial membrane potential also leads to degradation of Mfn1 and Mfn2, which mediate MOM fusion, and small GTPase MIRO, which drives mitochondrial movement (Tanaka et al., 2010; Wang et al., 2011). Eventually, the uncoupled mitochondria lose the fusion mechanism of MIM and MOM and continue to be isolated until removed by mitophagy, thus avoiding contamination of the healthy mitochondrial network.

Mitochondrial fission affects the number of mitochondria in a cell. During the G1/S transition of cell cycle, cyclin dependent kinase (CDK), which mediates the synthesis of new chromosomes, activates DRP1 and stimulates mitochondrial fission, so as to ensure that there are adequate mitochondria in the daughter cells produced by cell division (Salazar-Roa and Malumbres, 2017). Moreover, mitochondrial fission is also a member of the mitochondrial quality-control system, which can update the organelle function by removing damaged organelle components (Tanaka et al., 2010). It has been reported that mitochondrial fission usually produces daughter Mitochondria with different membrane potential, one of which is hyperpolarized, while its sibling, conversely, is low-polarized (Twig et al., 2008). This asymmetric mitochondrial fission is caused by the asymmetric distribution or aggregation of damaged components or fragments in the mitochondria, so that the daughter mitochondria that inherit more harmful components or aggregates in the process of mitochondrial division are more likely to lose membrane potential and be cleared by mitophagy. This phenomenon is similar to the phenomenon that bacteria remove aggregates by asymmetric fission to increase the growth rate of daughter cells, which have no aggregates (Lindner et al., 2008).

In conclusion, mitochondrial fusion provides a method to dilute the concentration of mutant mitochondrial genes and reduce the incidence of mitochondrial dysfunction. Under stress, active mitochondrial fusion helps to maintain the stable production of mitochondrial ATP, and increases the cell’s resistance to injury through functional complementation between mitochondria, but severely damaged mitochondria are prevented from reintegrating into the mitochondrial network. Mitochondrial fission provides a “condensation” mechanism by which damaged components or aggregates of the organelles accumulate in some daughter mitochondria and are removed by mitochondrial autophagy.

## Mitophagy Scavenging Irreparable Mitochondria

Autophagy is a catabolic pathway that leads to lysosomal degradation of intracellular components, and non-specific autophagy of basal intensity is essential for the degradation of impaired and dysfunctional cellular components and organelles to maintain cell homeostasis. Autophagy clearance of mitochondria, or mitophagy, is the way for cells to eliminate aging mitochondria and act as a “gatekeeper” of the mitochondrial quality control system when mitochondria are severely damaged (Ma et al., 2020).

Mitochondria provide various metabolic support for cells, but the output of dysfunctional mitochondria represented by ATP is reduced. In extreme cases, F_1_F_0_-ATPase on the MIM may not only fail to produce ATP, but also consume intracellular ATP to maintain mitochondrial membrane potential (Buchet and Godinot, 1998). Under physiological conditions, ROS produced by mitochondria is an important signaling substance and can be regulated to moderate level through endogenous antioxidant mechanism. However, when the mitochondria are defective, inaccurate ETC transmission significantly increases the production of ROS and makes the scale of ROS out of control, thereby accelerating oxidative damage and even directly leading to apoptosis or necrosis. Moreover, mitochondrial contents (e.g., mtDNA, N-formyl peptides and mitochondrial lipids represented by cardiolipin), released as damage associated molecular patterns (DAMPs) when mitochondria are damaged, can be identified by the innate immune system, which arose evolutionarily in primitive eukaryotes that innately recognize pathogen associated molecular patterns (PAMPs) of viruses and bacteria (Zhang et al., 2010; Rodriguez-Nuevo et al., 2018). Activation of innate immune pathways by DAMPs can lead to various forms of inflammatory damage to adjacent cells and even distant tissues (Galluzzi et al., 2012a).

Identifying severely damaged mitochondria is the core of this quality control process, and abnormal mitochondrial membrane potential serves as a sensitive target of mitophagy (Figure 2). In healthy mitochondria, the kinase Pink1 is degraded by protease PARL after being transported to the mitochondria (Narendra et al., 2008). When the mitochondria is severely damaged, the loss of mitochondrial membrane potential prevents Pink1 from entering the mitochondria and being degraded. Pink1 aggregates on the mitochondrial TOM complex and phosphorylates ubiquitin binding to damaged proteins that have not yet been degraded by the proteasome. Phosphorylated ubiquitin recruits and activates the E3 ligase Parkin, which ubiquitinates more proteins, and interacts with Pink1 to form a feed-forward loop mechanism (McWilliams and Muqit, 2017). These ubiquitin chains bind several receptors like optineurin, NDP52 and p62, which then initiate the mitophagy and culminates by releasing mitochondria into the lysosome for degradation (Lazarou et al., 2015; Khaminets et al., 2016).

In addition to transcriptional up-regulation of non-specific autophagy, many stress conditions, such as nutrient deprivation, ischemia, hypoxia, glycogen depletion, or exposure to cytotoxic agents can also directly affect specific mitophagy (Kaur and Debnath, 2015). For example, decreased availability of intracellular ATP during hypoxia leads to the increase of AMP/ATP ratio and the activation of AMP-activated protein kinase (AMPK). The phosphorylation of mitochondrial fission factors (MFFs), which depends on the activation of AMPK, enables mitochondria to recruit DRP1 from the cytoplasm, thereby promoting the mitochondrial fission and subsequent mitophagy of severely damaged mitochondria (Toyama et al., 2016). This also suggests that though autophagy is up-regulated relative to baseline during stress, its target still remains selective and tends to engulf suboptimal components. This process breaks down cellular components, such as proteins, lipids, glycogen, and ferritin, to help sustain and facilitate core anabolic and biosynthetic fluxes in cells under stress, while also improving the quality of the mitochondrial population (Suliman and Piantadosi, 2016).

## Apoptosis Activation to Intercept Uncontrolled Cell Death

**FIGURE 3:**
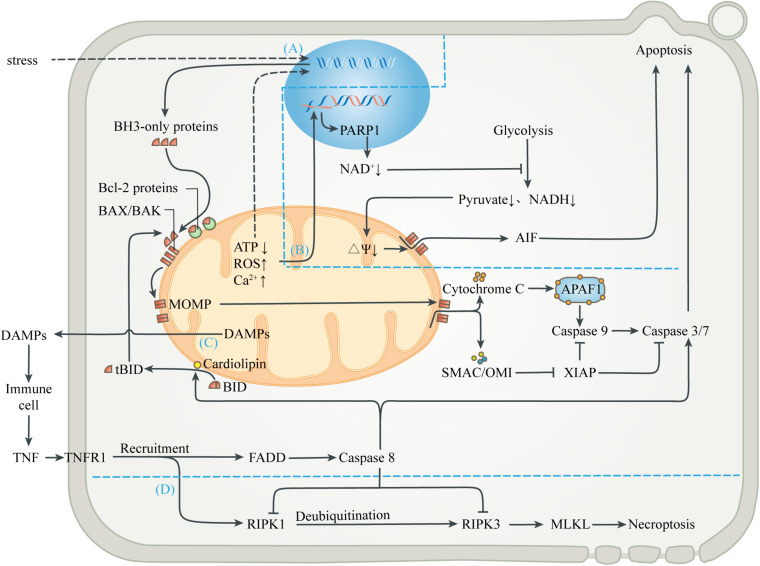
Mitochondria-mediated regulated cell death. **(A)** Mitochondrial apoptosis. Signals from mitochondrial injury stimulate the synthesis of BH3-only proteins, which can activate BAX/BAK on mitochondrial membrane to cause MOMP when the isolation of anti-apoptotic Bcl-2 proteins is exceeded. Cytochrome C interacts with APAF-1 to form the heptameric apoptosome, which initiates the caspase cascade to execute apoptosis. Activation of caspases can be inhibited by XIAP, which in turn is blocked by SMAC and OMI. Activated caspases 3 and 7 cleave cell components, including nuclear DNA and ultimately cause apoptosis. **(B)** Parthanatos. Mitochondrial abnormalities can also directly lead to nuclear DNA damage, which activates PARP1 to deplete NAD^+^, thereby inhibiting glycolytic production of pyruvate and NADH. In the absence of substrates, mitochondrial depolarization triggers the release of AIF, leading to parthanatos. **(C)** Exogenous apoptosis. The release of contents during mitochondrial injury activates immune cells and leads to the release of TNF. After TNF binds to TNFR1, FADD is recruited and caspase 8 is activated, directly activating caspase 3/7 to induce apoptosis. Caspase 8 can also activate BID to enter the mitochondrial apoptosis pathway. In this process, cardiolipin provides an anchor and an activating platform for caspase-8 and BID at the mitochondrial membrane surface. **(D)** Necroptosis. RIPK1 is also recruited after the binding of TNF and TNFR1, but recruited RIPK1 is usually ubiquitinated and inactivated. ROS, acidosis and other factors can lead to deubiquitination of RIPK1, which in turn leads to phosphorylation of RIPK3 and MLKL, leading to interruption of membrane continuity and necrosis. However, in most cases, simultaneous activation of caspase 8 can lyse RIPK1 and RIPK3 and truncate the Necroptosis.

Mitochondria are important sensors of eukaryotic cells, where danger signals caused by disturbance of homeostasis can converge. Under relatively mild stress, adaptive responses in the mitochondria help to restore homeostasis. Conversely, when the damage is irreversible and exceeds the cell’s compensation, the release of substances in mitochondria caused by danger signals can be a means of inducing apoptosis (Galluzzi et al., 2012a; Figure 3). Apoptosis, when ATP is still present and cells have time to respond, almost always takes precedence over necrosis, a more uncontrolled form of cell death, thus minimizing damage to external tissues caused by the release of mitochondrial contents due to cell membrane rupture (Hotchkiss et al., 2009; Vakifahmetoglu-Norberg et al., 2017).

The mechanism of apoptosis may be derived from the endosymbiotic relationship between alpha-proteobacterium and eukaryotic cell precursors, and developed with the evolution of endosymbiont (Kroemer, 1997). The presence of CED-9 (ortholog of human BCL-2) in the MOM of *Caenorhabditis elegans* is one of the earliest indications that mitochondria are involved in cell apoptosis (Hengartner et al., 1992). Since then, numerous studies have revealed the molecules and pathways involved in the initiation and execution of regulated cell death.

Various stresses, including excessive ROS, calcium overload, nutritional deficiencies, chemoradiotherapy, and cytotoxic agents, can induce endogenous apoptosis (also known as mitochondrial apoptosis pathway) (Singh et al., 2019). Many studies have demonstrated that, a decrease in mitochondrial transmembrane potential, followed by mitochondrial uncoupling and generation of ROS, precede nuclear alterations during apoptosis (Vayssiere et al., 1994; Kroemer et al., 1995; Zamzami et al., 1995). Intracellular elements (e.g., transcription factors and co-mediators) undergo conformational changes after sensing the stress stimulation, and incorporate into the nucleus to activate upstream genes (e.g., p53, MYC), thus up-regulating pro-apoptotic proteins (e.g., BIM, PUMA, tBID) (Sarosiek et al., 2017). When the isolation effect of pro-survival proteins (e.g., BCL-2, BCL-X_L_, or MCL1) is exceeded, these pro-apoptotic proteins activate the membrane opening proteins BAX and/or BAK, causing MOM permeability (MOMP), through which cytochrome C, SMAC and OMI are released into cytoplasm from the MIMS (Wensveen et al., 2010; Singh et al., 2019). Cytochrome C released from mitochondria interacts with APAF-1 to form the heptameric apoptosome, which initiates the caspase cascade to execute apoptosis (Yadav et al., 2020). Activation of caspases can be inhibited by the released XIAP, which in turn is blocked by SMAC and OMI. Activated caspases 3 and 7 extensively cleave cell components, including nuclear DNA and ultimately cause caspase-dependent apoptosis (Julien and Wells, 2017; Dorstyn et al., 2018). In addition, other mitochondrial proteins, such as endonuclease G and Omi/HtrA2, were found to undergo release during apoptosis and have been implicated in various aspects of the cell death process (van Loo et al., 2002). On the one hand, apoptotic regulators and executioners are involved in non-lethal physiological processes, such as cell cycle progression, differentiation, metabolism, autophagy and inflammation (Galluzzi et al., 2012b).

Mitochondria also participate in the apoptosis induced by extracellular stimulation, namely exogenous apoptotic pathways (also known as death receptor apoptotic pathways). DAMPs released extracellular during mitochondrial injury activates surrounding innate immune cells, such as neutrophils and macrophages, which can release TNF and Fas-L as sources of death signals. The initiation of exogenous apoptosis involves the binding of death receptor ligands (e.g., TNF-α, Fas-L, TRAIL) with the death receptor family (e.g., TNFR1, Fas, TRAILR) (Wang and Su, 2018). For example, TNF-α binds to TNFR1 and polymerizes FADD to form a death complex through the death domain of recruited adapter TRADD. This complex leads to oligomerization and activation of the initiator caspase 8, which in turn cleaves and activates the executor caspase 3 and 7, leading to apoptosis. In addition, activated caspase 8 can also cleave the BH3-only protein BID into truncated BID (tBID), which is then transferred to the MOM and activates the endogenous apoptotic pathway (Bock and Tait, 2020). In this process, cardiolipin has been shown to provide an anchor and an essential activating platform for caspase-8 and BID at the mitochondrial membrane surface, thus bridging the gap between death receptors and mitochondria (Jalmar et al., 2013).

Different from caspase-dependent apoptosis, parthanatos uses apoptosis-inducing factor (AIF), a NADH oxidoreductase needed to stabilize the respiratory complex, as the executor of cell death (Galluzzi et al., 2012a). DNA damage induced by oxidative stress and cellular ischemia can activate a nuclear DNA repair enzyme, poly ADP-Ribose polymerase 1 (PARP1), which needs to cleave the ADP-ribose moiety from NAD^+^ during DNA repair. Excessive PARP-1 activation depletes intracellular NAD^+^ and inhibits glycolysis, which supplies pyruvate and NADH equivalent to mitochondria (Alano et al., 2010). Mitochondria lacking metabolic substrates suffer from the loss of MIM potential, which promotes MOMP and triggers the release of AIF (Alano et al., 2004). AIF enters the nucleus and causes DNA fragmentation and degradation, leading to cell death.

In addition to regulating apoptotic cell death, mitochondria are also associated with necroptosis, which is a form of regulated necrosis (Vandenabeele et al., 2010; Vitale et al., 2011). Studies have shown that the activation of death receptors can recruit and form RIPK1-RIPK3 complex (i.e., necrosome) after some stimulation, transmit necrotic signals, activate MLKL, and ultimately leads to continuous interruption of mitochondrial membrane and necrosis (Galluzzi and Kroemer, 2011). The initiation of necroptosis is related to oxidative and metabolic burst and acid-base disorders, which are mainly regulated by mitochondria (Vandenabeele et al., 2010). Interestingly, the upstream pathway leading to necroptosis can also initiate exogenous apoptosis (Wang et al., 2014; Ni et al., 2019). Activated caspase 8 can cleave RIPK1 and RIPK3, and thus prevent necrotic cell death (Galluzzi et al., 2011; Yuan et al., 2019). The abortion of necrotic cell death caused by apoptotic activation also marks the evolutionary selection of a more optimal death strategy in eukaryotes.

Necrosis can also be observed in the late stage of apoptosis, which is called secondary necrosis. When the phagocytic defect or immunosuppression leads to insufficient phagocytic capacity of apoptotic cells, the uncleaned corpses may rupture in the form of necrosis, causing inflammation and autoimmune injury (Hochreiter-Hufford and Ravichandran, 2013; Martins et al., 2017). To ensure timely phagocytosis, the dying cells send out “find-me” signals to attract phagocytes, including lysophosphatidylcholine, sphingosine-1-phosphate and nucleotides (Ravichandran and Lorenz, 2007; Hochreiter-Hufford and Ravichandran, 2013). Phosphatidylserine, which is exposed to the outer surface of cell membrane during apoptosis, acts as a “eat-me” signal to guide phagocytes to engulf apoptotic cells (Suzuki et al., 2013).

In conclusion, when severe stress exceeds the mechanism of cellular compensation and repair, eukaryotic cells can preferentially activate various apoptosis pathways, so as to truncate necrosis and avoid mitochondrial DAMPs and ROS release that cause huge damage to surrounding cells or even distant tissues.

## Conclusion and Perspective

Over 1.5 billion years of obligate endosymbiosis and co-evolution between anaerobic alpha-proteobacterium and eukaryotic cell precursors have made this pair stand out in natural competition. The cooperation between eukaryotic cells and mitochondria has greatly improved the efficiency of metabolic substrates to produce ATP and increased the cell biosynthesis flux, which is the basis of single-cell evolution and multicellular development of eukaryotes (Aktipis et al., 2015; Otten and Smeets, 2015; Trosko, 2018). However, every coin has two sides. The increased metabolic requirements associated with complex cellular structure and function can no longer be met by inefficient glycolysis, increasing the dependence of eukaryotic cells on mitochondria. Therefore, eukaryotes need to continuously optimize the function of mitochondria and adjust the output of mitochondrial according to demand. The advent of multicellularity also made the mitochondrial quality control in eukaryotic cells not only based on the needs of individual cells, but also subject to the interests of multicellular organism.

Mitochondrial function and behavior are central to the physiology of humans and consequently mitochondrial dysfunction is implicated in a wide range of diseases, encompassing all aspects of medicine. The processes that regulate and optimize mitochondrial function in many non-human eukaryotes are becoming clearer, but how these pathways work in the variety of human cells with very different energy and metabolic demands remains elusive. Disruptions to mitochondrial homeostasis caused by excessive damage or inadequate repair lead to a variety of human diseases, many of which have muscular or neuronal symptoms, and understanding how failures in mitochondrial maintenance lead to human diseases is in its infancy. The development of animal models that faithfully simulate human mitochondrial diseases is essential to understand the physiological significance of these pathways, to elucidate the highly tissue specific functions and regulation of mitochondria, and to develop therapeutics (Johnson et al., 2007). In addition, the lack of non-invasive or minimally invasive assessment methods for mitochondrial structure and function, such as imaging and hematological examinations, also limits the development of mitochondrial therapy (Murphy and Hartley, 2018; He et al., 2020). Recent technological developments will allow for system-based biochemical, metabolic and genomic approaches, which will provide valuable insight into mitochondrial biology. These approaches will help to construct a complete map of mitochondrial network that will be valuable for understanding the role of mitochondrial dysfunction in human diseases. There is no doubt that the subtle relationship between eukaryotic cells and mitochondria, and the interplay between endosymbionts and the whole organism, are far more complex than are currently appreciated. The future progresses of endosymbiosis research will definitely push mitochondrial therapy further to the bedside.

## Author Contributions

MZ performed literature searches and wrote the manuscript. HW and JY conceived the idea and supervised the writing process. YH and HD gave suggestions and refined the manuscript. All authors contributed to the article and approved the submitted version.

## Conflict of Interest

The authors declare that the research was conducted in the absence of any commercial or financial relationships that could be construed as a potential conflict of interest.
